# Robot Assisted Gait Training in a Patient with Ataxia

**DOI:** 10.3390/neurolint14030045

**Published:** 2022-06-22

**Authors:** Gianfranco Lamberti, Gianluca Sesenna, Martina Marina, Emanuela Ricci, Gianluca Ciardi

**Affiliations:** 1 Spinal Unit, Azienda Usl, 29121 Piacenza, Italy; g.lamberti2@ausl.pc.it (G.L.); emanuela.ricci@unipr.it (E.R.); 2 Degree Course of Physiotherapy, University of Parma-Piacenza Training Center, Viale Abruzzo 12, 29017 Fiorenzuola d’Arda, Italy; 3 U&O, 29017 Fiorenzuola d’Arda, Italy; gianluca.sesenna@uando.it; 4 Degree Course of Physiotherapy Student, University of Parma-Piacenza Training Center, Viale Abruzzo 12, 29017 Fiorenzuola d’Arda, Italy; martina.marina1@studenti.unipr.it

**Keywords:** ataxia, robotic-assisted gait training, rehabilitation, exoskeleton

## Abstract

Background: Ataxia is a neurological sign characterized by motor coordination during gait/voluntary limb movements impairment. Ataxic gait leads to disability and worsening of quality of life; physiotherapy intervention is recommended to improve motor function. Recent studies showed benefits due to repetitive robotized assisted gait training using a static exoskeleton in patients affected by acquired ataxias. The aim of the study was to perform a preliminary evaluation of the short-term effects of overground UAN.GO^®^-assisted gait training in an adult patient with ataxia but with no clear genetic pattern. Methods: This case report study was conducted on a single male adult patient, who presented ataxic spastic gait, posterior chain tightness, pes cavus, and unstable standing position. The patient underwent two preliminary sessions to take part in the study. Treatment protocol planned 10 sessions and each one lasted 80 min, 60 of which were spent in gait training using the mobile overground exoskeleton UAN.GO^®^. At T1 (start of the study) and T10 (final evaluation) assessments using the Scale for the Assessment and Rating of Ataxia, Berg Balance Scale, 6-Minute Walking Test, and Likert Scale were administered. Space-time parameters of gait cycle were also evaluated: left and right step length, stance and swing percentages. Results: improvements on the Scale for the Assessment and Rating of Ataxia, Berg Balance Scale, and in the distance travelled at 6-Minute Walking Test emerged. The patient gave a positive opinion towards the treatment, showed by Likert Scale results. Kinematic gait analysis showed more physiological step length, stance and swing percentages, joint angles. The patient completed the training program with an excellent compliance. Discussion: Since these encouraging outcomes were obtained, it is possible to consider robot-assisted gait training performed with UAN.GO^®^ as a therapeutic option to improve motor and functional performance in patients with ataxic gait.

## 1. Introduction

Ataxia (Greek *ataxia* “without order, coordination”) is a clinical neurological sign characterized by motor incoordination [1], which occurs in a wide range of diseases, involving cerebellum dysfunctions or impaired vestibular/proprioceptive input to cerebellum [2]. There is more than one strategy to classify ataxia: by age of onset, clinical course, anatomic lesion, and genetic/acquired origin [2]. Among inherited ataxias, dominant (spino-cerebellar ataxias—SCA, Dentato-rubral-pallido-luysian atrophy, and episodic ataxia) and recessive (Friedriech, teleangectasia, oculomotor, Refsum, and mitochondrial forms) autosomal forms have been staged [2,3]. Acquired forms, instead, result from acute or chronic conditions such as strokes, multiple sclerosis, infections, vestibular neuritis, toxic disorders, and immune deficiency syndromes [2,3,4].

When ataxia presents as a neurological sign of a greater disease, a wide range of non-motor symptoms are present; this commonly occurs in hereditary syndromes [5]. Data about ataxia syndromes’ epidemiology show that overall prevalence is 26 cases per 100.000 in a young population; in detail, hereditary ataxia affects 10 people out of every 100.000, with higher rate for dominant types (2.7/100.000 versus 3.3/100.000 for recessive forms); non-hereditary forms have prevalence of 4.9/100.000 [6,7].

From a clinical point of view, each body district can be affected by ataxic tract, as well as muscular structures: limbs, trunk, bulbar, and ocular movements [3]. Ataxia of limbs is characterized by a variable combination of dysmetria, dyssynergia, dysdiadochokinesia, kinetic, and/or postural tremor. Ataxia of trunk is represented by the inability to maintain sitting position; ataxia of speech by cerebellar dysarthria; ataxia of gaze by nystagmus and ocular dysmetria [3]. Ataxic gait is typically characterized by unstable walking, increased step width, and balance deficit and includes the following space-time features:

- reduced cadence and speed
- reduced step and stride length
- increased stance time
- increased step and stride time
- increased double limb support time
- increased step/stride length variability and increased stride time variability, which represent a compensatory strategy for trunk instability [8].

These gait modifications cause a loss of independence in activities of daily life and falls.

Robotic rehabilitation, based on continuous assessment of patients’ progress, can be useful to complete ataxia conservative management [9]. In particular, lower limb exoskeletons, recently developed, are amongst the most promising rehabilitative means of robot-assisted gait training (RAGT). They are self-supporting and make patients carry out pre-programmed physiological gait cycles. RAGT has important advantages compared to the traditional gait training: increase of training duration, improving gait quality, and reducing the patient’s and therapist’s physical effort [10,11]. Use of exoskeletons in neurorehabilitation is based on the hypothesis that such devices exploit plasticity [12], are activity-dependent and use-dependent, and are essential for motor learning [13,14]. However, evidence on RAGT effectiveness in ataxic patients is barely summarized and attributable to a preferential treatment protocol. The aim of the study is to provide preliminary evidence on RAGT using UAN.GO^®^ overground exoskeleton in an adult patient with ataxia.

## 2. Materials and Methods

A single male subject, 56 years old, with a diagnosis of ataxia was recruited for this case report. Inclusion criteria: adult age, strong motivation, eligibility to use UAN.GO^®^ exoskeleton. Exclusion criteria: psychiatric and cognitive disorders and spasticity (scoring more than 3 on Ashworth scale). No ethics committee approval was required, as suggested for clinical case reports by AVEN guidelines [15].

The patient signed informed consent before the treatment.

Familiar, physiologic, personal, and pharmacologic history was irrelevant; his diagnostic pathway started since the childhood, with the onset of increasing walking and balance difficulties. He was visited by an Italian reference center for rare diseases, and his final diagnosis was “Friedrich’s ataxia, characterized by poorly progressive gait.” In 1983 he underwent EMG investigation, which reported signs of suffering of upper and lower limbs. In 2007, with genetic sampling, pathological expansion of the FXN gene was ruled out (thus excluding Friedrich ataxia), and geneticists hypothesized the presence of a de novo mutation that had not yet been staged. Only in 2022, our patient has been newly contacted by the referral center and he began the process of reclassifying his pathology, which is still ongoing.

Other features about his past medical history: subtrochanteric fracture of left femur after an accidental fall during skiing in 1996 and a head trauma caused by a motorcycle accident in 2001.

At the start of the study, the patient was able to walk with 2 Nordic Walking sticks (used since 2016), with a crossed walking scheme; physical examination showed bilateral pes cavus, dorsal kyphosis, and ataxo-spastic gait, characterized by a scissor scheme, flexion-adductor pattern, wide base of support, asymmetric left and right step, balance deficit, and progressive increase of gait speed. Muscle tone of hamstrings, triceps surae, and posterior kinetic chain was lightly increased (Ashworth score = 1). There were also widespread hyperreflexia and bilateral extensor plantar responses. Muscular length test showed shortening of posterior and anterior inferior kinetic chain. Romberg test both with open and closed eyes was positive. The subject was also unable to maintain tandem position and single leg stance. Nose-finger test was lightly positive with an amplitude of intentional tremor <2 cm in both upper limbs. At the finger chase, the subject showed light dysmetria with overshooting target <5 cm. Fast alternating hand movements were performed in a slight and irregular way (10 cycles in less than 10 s); heel-shin slide was lightly abnormal and there was also tremor, but the contact between shin and heel was maintained. The subject had light dysarthria with scanning speech. No visual, auditory, or swallowing abnormalities emerged. The subject was completely independent in basic and instrumental activities of everyday life.

Treatment was carried out through innovative UAN.GO^®^ exoskeleton for lower limbs, designed by U&O s.r.l., MedTech company, whose aim is to propose innovative therapeutic solutions to improve life quality in people with motor disabilities. U&O is currently the first Italian company to have a certified active exoskeleton. UAN.GO^®^ is a motorized exoskeleton for the Overground robotic gait training (O-RAGT), which allows people with motor disabilities to walk in an independent way, moving themselves in the space. UAN.GO^®^ was certified as medical device (CE IIA) for clinical and personal use; the device is equipped with four motorized joints (hips and knees) and four passive joints (ankles and feet). They allow an effective gait training thanks to advanced sensors and innovative control strategies. UAN.GO^®^ is a self-supporting exoskeleton and its weight is completely discharged to the ground; this device is intended for individuals with complete SCI at levels T4 to L5 (with upper extremity motor function of at least 4/5 in both arms) and incomplete SCI at levels of C7 to T3 (with upper extremity motor function of at least 4/5 in both arms) and is also designed for patients with hemiplegia due to stroke, multiple sclerosis, and Parkinson’s disease; the training can be performed in all walking disabilities. There are two modes of use: “Assisted Mode” when the caregiver selects the movement map from the touchscreen and pushes start/stops buttons. In “Autonomous Mode” map, the patient actively controls the exoskeleton through trunk movements; firstly, in Autonomous Mode the caregiver has to set up the trigger position. Therapy is constantly adjusted and personalized on different levels of motor ability and patient’s needs; power support can be partial or total, depending on residual motor function. For this reason, UAN.GO^®^ exoskeleton allows the patient to perform robotized intensive training and the therapist to reduce musculoskeletal overload, carry out an intense and effective training and quantify specific training data.

The subject needs to meet specific inclusion criteria, represented in Table 1, in order to be suitable to the training; some general contraindications have to be excluded, as for other robotic devices (Table 1).

**Figure 1:**
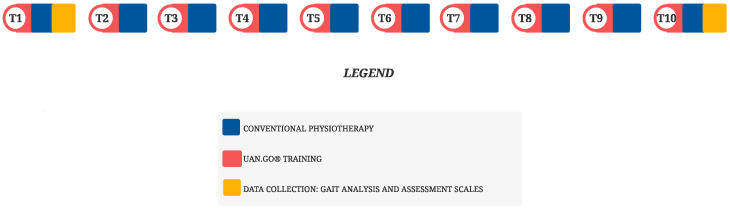
Timeline of RAGT/physiotherapy intervention; the patient was initially assessed for eligibility to the study (T1) with SARA score, Berg balance scale, 6MWT, Likert scale. A parallel program of ten session of flexibility exercise and RAGT training was than performed by the same physiotherapist. The last session (T10) was dedicated to the final evaluation.

The patient underwent two preliminary-evaluation sessions and 10 treatment sessions (Figure 1); each one lasted 80 min and was performed twice a week. Each session was divided in five phases: stretching (10 min), software setup (2 min), mechanical-anatomical setup (3 min), gait training (60 min), and autonomous walk with aids (5 min).

### Therapeutic Intervention

The parameters speed and map of movement were adjusted during the treatment, depending on the patient’s progressive abilities and requests. All training sessions were performed in U&O location under a physiotherapist’s guidance; each training session started with ten minutes of stretching exercise for posterior muscular chain.

**Figure 2:**
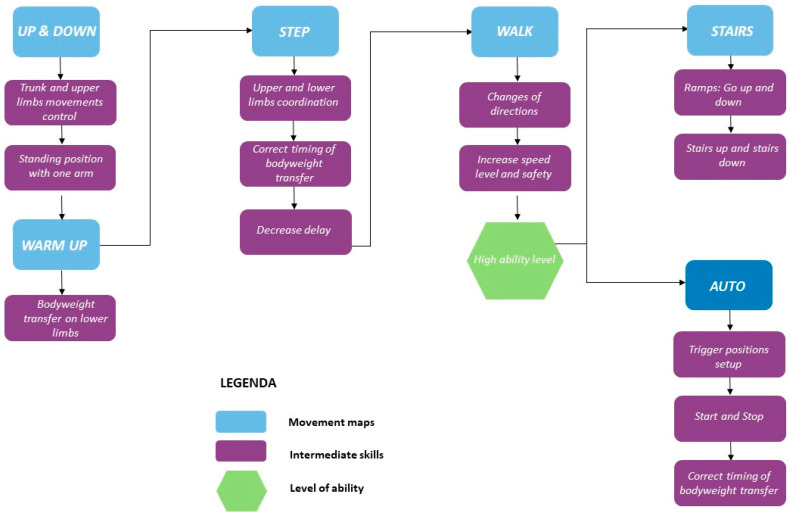
UAN.GO^®^ protocol; the training was divided in two moments: during pre-walk training four abilities were gained: up and down (sitting and standing), step (load transfer), walk (acquisition of physiological gait phase), stairs (climbing up and down ramps). With all abilities acquired, the patient started an intensive walk phase, to achieve the best change in step and swing parameters.

Robotized path for gait training was set with a specific approach to walking, based on literature evidence [16,17,18]. The protocol’s aim was to stimulate the patient in a progressive and proportional way. This training protocol was divided into two phases: PRE-WALK and WALK. The first one represents the starting point of treatment, and is aimed to learn baseline functions, preparatory to gait; the WALK phase is the most significant map from a rehabilitative point of view, and consists of real walking training. During WALK, the patients moves himself in the space and transfers load from a leg to other. Training protocol was briefly represented in Figure 2. During WALK training, the patient learns specific motor skills: coordination of different gait phases, improving of gait safety and speed, performing a correct gait timing, managing delay between left and right steps, and performing changes of direction. General training protocol was adapted to the patient’s needs and expectations; it was chosen to give more space to intensive training in WALK mode.

In T1 (study began) and T10 (final evaluation) specific assessment scales were administered: Scale of Assessment and Rating of Ataxia, Berg Balance Scale; walked distance in metres at 6-Minute Walking Test and space-time gait parameters (left and right step length, stance and swing percentages) were also considered. Space-time data were collected through videos made by the operator during subject’s self-paced walking on sagittal plane at 2.5 m distance. Videos were examined by the operator using Kinovea^®^ 0.8.15, a software of movement analysis in 2D available on the Internet for free, created in 2009; gait frames were so extracted to analyse changes.

## 3. Results

During five weeks of treatment, there were no side events; the patient carried out the rehabilitation program with an optimal compliance. Data collected in initial and final assessment were compared (Table 2).

SARA scale was administered by the physical therapist responsible of RAGT training, in order to evaluate changes in clinical features of ataxia [19,20]; as showed in Table 2, the total score in T1 was 11, while in T10 it was 9, showing an improvement in gait and stance subitems. In T10, gait performance was better, still staggering but in a slighter way than T1, with difficulties in half-turn but no more necessity of intermittent wall support. The subject showed his ability to maintain standing position with feet together (but not in tandem position) for >10 s. Scores of remaining subitems were unchanged.

BBS scale was used to evaluate balance function through 14 items, which ask the subject to maintain a specific position, to carry out specific motor tasks and postural transitions [21]. A significant difference (six points) between total BBS score in T1 and T10, higher than values of minimal clinically significant difference cited in literature, emerged at final evaluation [22,23]. An increase of single scores of BBS items was pointed out in:

- Sitting-standing position transfer: in T10, the subject was able to get up without the use of hands, and to stabilize himself in the new taken posture without any help.
- Standing-sitting position transfer: in T10, the subject could sit without danger of falling and with minimal use of upper limbs, without the need to control the drop with hands. In T1, the subject managed to sit with the help of upper limbs.
- Standing position with closed eyes: in T10, the subject could maintain a standing position with eyes closed, fixed for 10 s in safety. In T1, he could not perform the task and needed other person’s supervision.
- Picking up an object placed in front of the feet: in T10, the subject was able to pick it up in a safe and easy way. In T1, he could not pick it up alone.
- Rotating 360° in standing position: in T10, the subject could rotate completely and safely in a single direction in 4 s or less (pivot on left leg and left rotation). In T1, he could perform this movement very slowly.
- Put feet alternatively on a step during standing position: in T10, the subject could carry out more than two movements and needed minimal assistance. In T1, assistance was essential to prevent falls.
- Standing tandem position: in T10, the subject could put one foot in front of the other (not in tandem position) and maintain this position for 30 s. In T1, he could complete a small step.

For the patient’s functional status measurement [24], 6MWT was chosen; a significant increase of walked distance at 6MWT, from 144.76 m in T1 to 160.27 m in T10; 15.51 m is referred as minimally clinically significant difference defined by Bohannon et al. [25].

**Figure 3:**
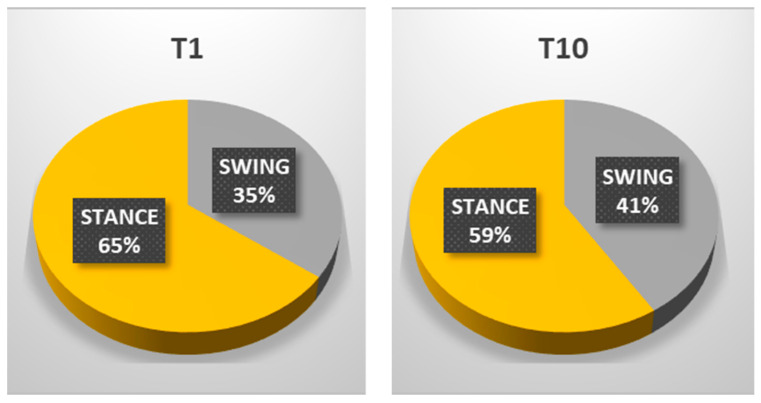
Differences in step percentages between initial (**T1**) and final evaluation (**T10**); as shown, initially the patient had a general prevalence of stance (65% of gait cycle). After RAGT and physiotherapy the stance was reduced to 59%, and swing phase was increased to 41%. These values are close to referred healthy subject ones (60% stance, 40% swing, Parry).

**Figure 4:**
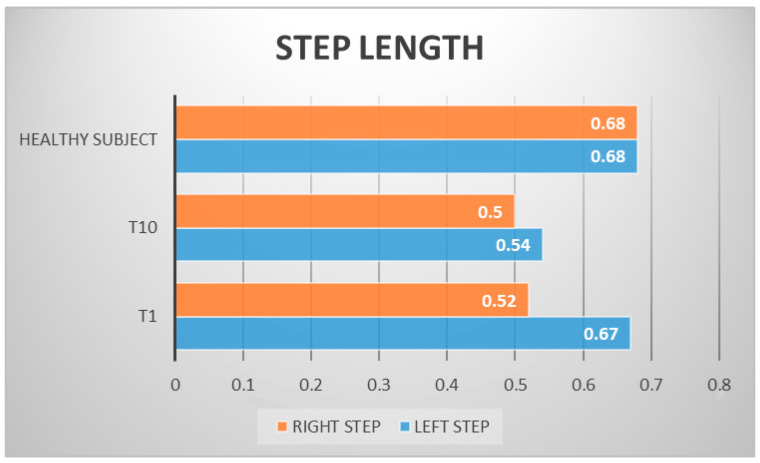
Left and right step length differences between initial and final evaluation; as shown, the patient had, at first, an asymmetrical gait pattern, with a delta left/right leg step of 15 cm. At final evaluation, this difference was diminished to 4 cm; this improvement shows that one of RAGT training’s advantages is the reaching of a symmetrical movement pattern.

**Figure 5:**
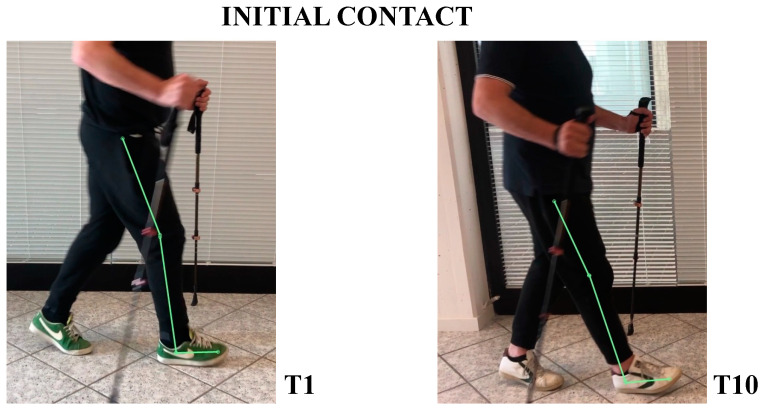
Initial contact frames at initial (**T1**) and final (**T10**) evaluation; with RAGT training, the patient gained a better alignment of the trunk and lower limb, with the onset of a real “heel strike” to the ground.

**Figure 6:**
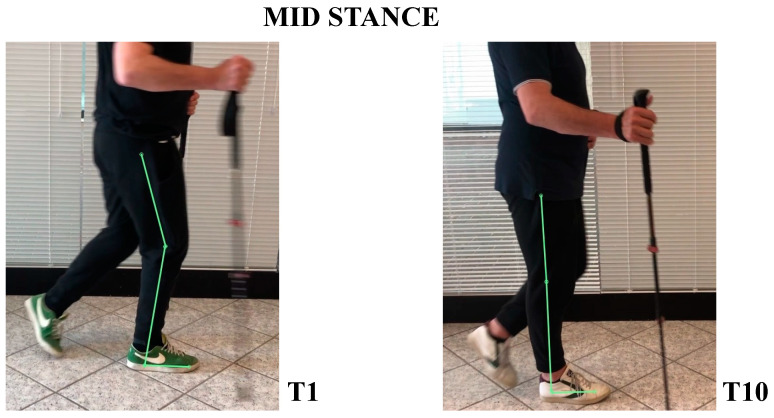
Mid-stance frames at initial (**T1**) and final (**T10**) evaluation; with RAGT training, the patient gained the ability to extend the loading knee and hip, allowing the swinging limb a more dynamic and less wasteful flexing movement.

**Figure 7:**
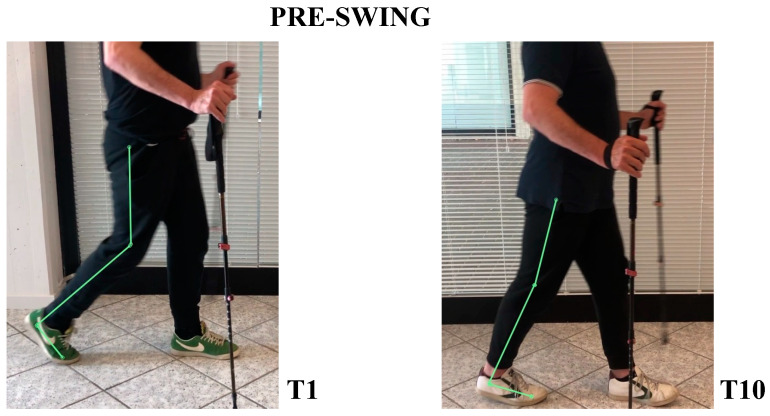
Pre-swing frames; with RAGT training, the patient became able to align foot-knee hip axis, thus allowing a greater propulsion to beginning step.

**Figure 8:**
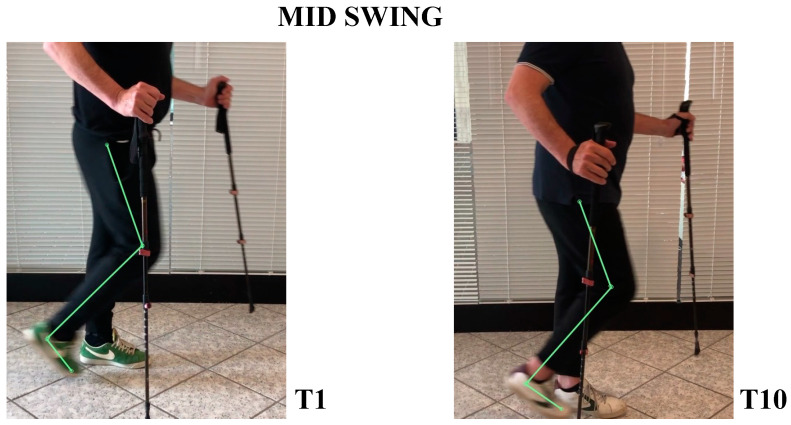
Mid-swing frames between initial and final evaluation. It is possible to see, during limb advancement, a better alignment in flexion, with a lower ankle angle to the ground. This improvement allowed the patient to walk with safety in everyday life.

The patient’s subjective point of view about training was evaluated through Likert Scale; he expressed a positive opinion towards this innovative approach to rehabilitation. The patient knew exoskeleton as a therapeutic solution and its continuous calibration; he also referred to an improvement of balance and stability during walking, and a better motor control of both lower limbs after a single treatment session. Kinematic gait analysis revealed improvements of time percentages of stance and swing phases. In T1, respective percentages of stance and swing were: stance 65.54%, swing 35.46%. In T10: stance 59.01%, swing 40.99% (Figure 3). Regarding the ratio between left and right step lengths: in T1, this ratio was almost 1.28, while in T10 it reduced to 1.08 (Figure 4). Regarding hip, knee, ankle ROM, and trunk and pelvis behaviour, in T1:

1. In initial contact phase, trunk was flexed, hip and knee were flexed; forefoot, midfoot, and backfoot touched the ground at the same time (Figure 5).
2. In mid-stance phase, trunk, hip, and knee flexion persisted, while ankle was dorsiflexed (Figure 6).
3. In the pre-swing phase, hip was in a neutral position on the sagittal plane, knee was flexed, and foot was equinus (Figure 7).
4. In the mid-swing phase, hip was slightly flexed and knee had a marked flexion to compensate for the forefoot fall in equinus (Figure 8).

In all phases, there was no control of the pelvis, with continuous tilting and rotation movements. The trunk was bent forward to promote propulsion.

In T10:

1. At initial contact, hip was flexed, knee was extended, and foot performed “heel strike” on the ground with slight ankle dorsiflexion (Figure 5).
2. In the mid-stance phase, hip and foot were in neutral position, while knee was extended (Figure 6)
3. In the pre-swing phase, hip went to extension, knee reached very few degrees of flexion, while foot was in dorsal flexion (Figure 7)
4. In the mid-swing phase, hip and knee were flexed, foot maintained neutral position with a reduced fall of the forefoot compared to the initial evaluation (Figure 8)

The trunk reached the neutral position, reducing anterior positioning; tilting and rotation pelvic movements were reduced and controlled.

## 4. Discussion

This case report was compliant with CARE guidelines [26]. The universal feature associated with all the forms of degenerative genetic ataxia is the loss of function and mobility [27]. Among currently available therapeutic options, rehabilitation assumes a huge importance; in particular, it is widely recognized that, through movement assistance provided by an exoskeleton, both the muscular and nervous system are stimulated where they are deficient, and thus predict an improvement in impaired function [28]. Movements produced by an exoskeleton not only represent an action, but also an idea, a new mode of motor planning [28] in which the intention has the same importance as the action. It is known that motivation, involvement [29], and positive patient feedback [30] are needed for rehabilitation success, to improve robotic technology as a resource [29].

Coming to our results discussion, the patient showed an improvement in the functional items of the SARA and BBS scales, relating to the static balance in standing, dynamic during postural transitions and walking; these improvements lead to hypothesize better motor control during walking, as well as a greater ability to carry out ADLs. The slight but significant increase in the distance covered at 6MWT probably reflects a better functional ability due to new convenient walking strategy. Step’s space-time parameters also highlighted a more physiological path, characterized by a better distribution of times in percentage of stance and swing and by greater symmetry of the right and left half steps. Thanks to robotic walking systems, patients could walk more fluidly with more harmonious relationships between different gait phases. From the frames obtained on the sagittal plane, in T10, a greater alignment of the trunk, better control of pelvic movements, and less support on the upper limbs emerged. At initial contact, the knee reached a greater degree of extension and the ankle joint reached a few degrees of dorsiflexion; all this allowed an adequate heel attachment. In the mid-stance phase, the knee maintained a greater degree of extension, and the hip reduced the angle of flexion until a neutral position was reached. In the pre-swing phase, the hip was brought into extension, poorly represented in T1, and the degree of knee flexion was reduced. During the mid-swing phase, the hip flexion angle was kept constant and the knee flexion angle was reduced. The overall performance of lower limbs improved at final evaluation.

Our results are in line with recent literature [28,31,32,33], encouraging RAGT treatment in patients with ataxia. Particularly, a close correspondence can be found with the work of Kim et al. [28], in which a patient with SCA6 underwent 24 sessions of training in the use of an exoskeleton; authors reported an improvement of the SARA scale, greater balance at the Berg scale, greater distance at the 10 MWT, and an improved gait cycle. All these findings are confirmed in our experience. Some similarities can also be found in the work of Portaro et al. [33]; the authors reported the case of a young patient with Friedrich’s ataxia treated with exoskeleton and TDCS (transcutaneous direct cranial stimulation). A clear improvement in SARA scores was also found in this case; it must be underlined, however, that this case report refers to an ataxic patient with not-full recognized origin and treated only with RAGT, so there is a comparison bias with Portaro et al.’s experience.

Similar effects of RAGT in neurologic chronic patients have been reached in recent RCTs, underlining the statistical significance of RAGT on trunk control evaluated by the Berg scale [32,33].

A final discussion on patient perspective about exoskeleton training is needed; our participant reported an excellent reference to the use of the exoskeleton, and a greater safety in performing daily life activities and walking, as detected by the Likert scale. These considerations seem to be yet in line with literature [28,31,32,33]. In light of these objective and subjective interpretations, it is possible to consider treatment with the UAN.GO^®^ exoskeleton as a viable therapeutic option in patients with ataxia.

## 5. Limitations and Conclusions

Some limitations emerged in the present work. Given the design of case report-study, we cannot generalize our results to all ataxia forms. Thus, the need to conduct further studies on patient cohorts, considering a non-homogeneous distribution of clinical manifestations, emerged. In addition, our study implemented a rehabilitation program of 10 sessions and provided data analysis/interpretation only for the short term; future studies are needed to verify the maintenance of results over time with systematic follow-up. We hypothesize that a longer treatment period allows an improvement of results. Limiting factors that most influenced the conduct of the study were the lack of equipment for collection and measurement of data and the rehabilitation setting. For the detection of space-time parameters of the step, we used Kinovea^®^; since it allows a gait analysis based on operator observation of video recordings, it does not produce objective data. Instrumental examination, which allows to analyse and record the path of a subject in a computerized way with optical and electronic systems, is Gait Analysis; today it is carried out in specific clinics that use infrared cameras capable of detecting passive markers positioned on the patient, or it can even be performed anywhere thanks to wireless sensors, also applied to the patient. To avoid obtaining an overabundance of data that would be difficult to analyse using this modality, we filmed the patient’s walk on the sagittal plane, on which the greatest articular excursions occur, omitting the minimum movements that occur on the frontal and transverse planes; the most significant frames, then, were selected and reported in the present article.

As for the training setting, internal spaces forced the patient to walk with the exoskeleton along straight paths, interrupting the WALK at each change of direction, which was carried out with an autonomous pivot with the help of the trunk and upper limbs. Space was a fundamental feature of robotic training with the exoskeleton; the availability of large and adequate spaces is necessary. Rehabilitation setting still represents an insurmountable limit, as long as times, costs, and spaces remain so.

Regarding the structure of UAN.GO^®^, the type of insole used facilitates the setting and positioning of the patient inside the device. This appendix limits the perception of proprioceptive inputs from the sole of the foot in patients in whom sensitivity is preserved, but at the same time allows the use of the device in a transversal way on patients with different alterations in motor function. Hence, there is a need to soon implement an additional solution consisting of footwear with an integrated insole, chosen by the clinician on the basis of the patient’s characteristics. In the field of robotic rehabilitation with an exoskeleton, human–robot integration acquires a central role in achieving better outcomes. Recent research topics are the study and application of adaptive active wearable technologies that compensate patients’ deficits in real time during the execution of a motor task [31].

In patients with ataxia, rehabilitation is aimed at maintaining the functional aspect and reducing symptoms and is recommended as a powerful therapeutic solution. This case report, although devoid of statistical significance, supports preliminary evidence of walking training with exoskeleton both from a motor and functional point of view.

## Figures and Tables

**Table 1:** Inclusion and Exclusion criteria to perform RAGT training.

Inclusion Criteria	Exclusion Criteria
Upper limbs able to handle a walker	History of severe neurological diseases associated with severe systemic diseases (e.g., infections, circulatory or heart problems, lung problems)
Absence of unconsolidated fractures	Presence of pressure sores
Good general health	Severe spasticity
Height between 155 and 195 cm	Heterotopic ossifications that reduce ROM
Weight not exceeding 100 kg	Spinal instability or pelvic or AAII fractures not healed
Good bone mineral density	Important retractions
	Psychiatric or cognitive problems that can interfere with the correct use of the device

**Table 2:** Comparison of T1-T10 outcome scores; as shown, the patient had lower impairment at SARA scale, a better balance control at BBS, improved distance at 6MWT, and a better distribution regarding gait parameters.

	T1	T10
**SARA**	**11**	**9**
Gait	**4**	**3**
Stance	**2**	**1**
Sitting	**0**	**0**
Speech disturbance	**1**	**1**
Finger chase	**1**	**1**
Nose-finger test	**1**	**1**
Fast alternating hand movements	**1**	**1**
Heel-shin slide	**1**	**1**
**BBS**	**41**	**47**
**WALKED DISTANCE IN 6MWT**	**144. 76 m**	**160. 27 m**
**LEFT STEP LENGTH**	**0.67 m**	**0.54 m**
**RIGHT STEP LENGTH**	**0.52 m**	**0.50 m**
**STANCE %**	**65.54%**	**59.01%**
**SWING %**	**35.46%**	**40.99%**

## Data Availability

Not applicable.
